# Beyond Knowledge: Addressing Health System Barriers to Hepatitis B Vaccination Among Healthcare Workers in Low‐ and Middle‐Income Countries

**DOI:** 10.1002/hsr2.73151

**Published:** 2026-08-26

**Authors:** Virak Sorn

**Affiliations:** ^1^ Faculty of Health Sciences and Biotechnology University of Puthisastra Phnom Penh Cambodia

Dear Editor,

I read with great interest the recent article by Yehualaw et al. reporting hepatitis B vaccination coverage and associated factors among healthcare workers (HCWs) in Ethiopia [1]. The authors should be commended for addressing an important occupational health challenge in a resource‐limited setting and for providing contemporary evidence on hepatitis B vaccination uptake among HCWs. Their finding that only 37.9% of HCWs had completed the recommended three‐dose vaccination schedule, despite relatively high levels of knowledge (69.9%) and favorable attitudes (79.9%), highlights a persistent disconnect between awareness and preventive practice. This observation has important implications not only for Ethiopia but also for many low‐ and middle‐income countries (LMICs), where occupational exposure to hepatitis B virus (HBV) remains a major public health concern.

While Yehualaw et al. identified infection prevention training, knowledge of HBV infection, knowledge of hepatitis B vaccination, and favorable attitudes as significant predictors of vaccine uptake [1], their findings also point to a more fundamental challenge: structural health‐system barriers may exert a greater influence on vaccination behavior than individual‐level determinants. Previous studies have consistently shown that HCWs are generally willing to be vaccinated but are frequently constrained by limited vaccine availability, financial barriers, weak occupational health policies, and inadequate institutional support [2, 3]. In the Ethiopian study, nearly all participants reported that hepatitis B vaccination was not provided free of charge by their institution, almost 60% perceived the vaccine as unaffordable, and cost was the most frequently cited reason for non‐vaccination [1]. Collectively, these findings suggest that inadequate institutional vaccine provision, rather than insufficient awareness alone, may represent the principal barrier to improving vaccination coverage.

This distinction carries important implications for public health policy. Educational interventions are essential for improving knowledge and shaping positive attitudes toward vaccination; however, knowledge alone rarely translates into sustained behavioral change when structural barriers remain unresolved. The well‐recognized “knowledge‐to‐action” gap in implementation science demonstrates that successful adoption of evidence‐based preventive interventions depends largely on the capacity of health systems to support implementation [4]. Consequently, expanding educational initiatives without simultaneously ensuring affordable and equitable access to hepatitis B vaccination is unlikely to substantially increase vaccine uptake among HCWs.

The reported association between infection prevention training and vaccination uptake also warrants cautious interpretation. Although HCWs who had received infection prevention training were significantly more likely to be vaccinated [1], the cross‐sectional design precludes causal inference. It is plausible that HCWs employed in institutions with stronger occupational health and safety cultures are both more likely to receive structured training and more likely to have access to vaccination services [5]. Likewise, healthcare professionals who are inherently more proactive regarding preventive health behaviors may be more likely to seek both professional training and vaccination. Therefore, the observed association may reflect broader institutional or behavioral factors rather than an independent effect of training itself.

The study also highlights broader policy implications extending beyond Ethiopia. The World Health Organization recommends hepatitis B vaccination as a core component of occupational health protection because HCWs face an elevated risk of exposure to blood‐borne pathogens [6]. Nevertheless, hepatitis B vaccination remains voluntary, inconsistently available, or self‐financed in many LMICs, creating avoidable inequities in occupational protection. Integrating hepatitis B vaccination into mandatory pre‐employment screening, comprehensive occupational health programs, and routine staff immunization services could substantially improve vaccine coverage while strengthening healthcare workforce safety [7]. These efforts should be supported by institutional monitoring systems that document vaccine completion, generate reminders for subsequent doses, and, where resources permit, incorporate post‐vaccination serological testing for HCWs at continued occupational risk.

Beyond individual and institutional determinants, future research should adopt a broader implementation science perspective by examining organizational and health‐system factors that were not fully explored in the current study. Variables such as vaccine procurement mechanisms, supply‐chain reliability, hospital leadership commitment, workplace safety culture, availability of occupational health services, and national immunization financing policies may substantially influence vaccination uptake. In addition, multicenter mixed‐methods studies incorporating qualitative approaches could provide valuable insights into contextual barriers and facilitators across diverse healthcare settings. Such evidence would better inform interventions that are both context‐specific and scalable across LMICs.

Overall, Yehualaw et al. make an important contribution by documenting persistently low hepatitis B vaccination coverage among HCWs despite generally favorable levels of knowledge and attitudes [1]. Their findings reinforce that improving vaccination coverage requires moving beyond educational interventions toward comprehensive, system‐level strategies that address affordability, accessibility, institutional accountability, and implementation capacity. Protecting HCWs from HBV infection should be viewed not merely as an individual responsibility but as a fundamental obligation of healthcare systems. Sustainable improvements in vaccination coverage will depend less on increasing awareness alone than on strengthening occupational health infrastructure, ensuring equitable vaccine access, and embedding hepatitis B vaccination into routine institutional practice. Such implementation‐focused strategies are essential for safeguarding the healthcare workforce and advancing progress toward the global elimination of hepatitis B.

## Author Contributions

**Virak Sorn:** conceptualization, writing – original draft, writing – review and editing.

## Funding

The author has nothing to report.

## Ethics Statement

The author has nothing to report.

## Consent

The author has nothing to report.

## Conflicts of Interest

The author declares no conflicts of interest.

## Data Availability

Data sharing is not applicable to this article as no datasets were generated or analyzed during the current study. Due to the fact that no data sets were created or examined for this article.
